# Disclosure, stigma of HIV positive child and access to early infant diagnosis in the rural communities of OR Tambo District, South Africa: a qualitative exploration of maternal perspective

**DOI:** 10.1186/s12887-015-0414-8

**Published:** 2015-08-26

**Authors:** Vincent Oladele Adeniyi, Elza Thomson, Daniel Ter Goon, Idowu Anthony Ajayi

**Affiliations:** School of Health Sciences, University of Fort Hare, East London, South Africa; African Centre for HIV/AIDS Management, Faculty of Economic Management Sciences, University of Stellenbosch, Stellenbosch, South Africa; School of Humanities and Social Sciences, University of Fort Hare, East London, South Africa; Adult Wellness/EMTCT Research Project, Cecilia Makiwane Hospital, East London Hospital Complex, Private Bag X 9047, Mdantsane, East London, 5200 South Africa

**Keywords:** Early infant diagnosis, HIV-exposed infants, Maternal perspectives, Rural, South Africa

## Abstract

**Background:**

Despite the overwhelming evidence confirming the morbidity and mortality benefits of early initiation of highly active anti-retroviral therapy (HAART) in HIV-infected infants, some children are still disadvantaged from gaining access to care. The understanding of the maternal perspective on early infant HIV diagnosis and prompt initiation of HAART has not been adequately explored, especially in the rural communities of South Africa. This study explores the perspectives of mothers of HIV-exposed infants with regard to early infant diagnosis (EID) through a lens of social and structural barriers to accessing primary healthcare in OR Tambo district, Eastern Cape Province, South Africa.

**Methods:**

In this qualitative study, we conducted semi-structured interviews at two primary healthcare centres in the King Sabata Dalindyebo Municipality of the OR Tambo district, South Africa. Twenty-four purposive sample of mothers of HIV-exposed infants took part in the study. Interviews were tape-recorded, transcribed and field notes were obtained. The findings were triangulated with two focus group discussions in order to enrich and validate the qualitative data. Thematic content analysis was employed to analyse the data.

**Results:**

The participants have fairly good knowledge of mother-to-child transmission of HIV and the risks during pregnancy, delivery and breastfeeding. The majority of participants were confident of the protection offered by anti-retroviral drugs provided during pregnancy, however, lack knowledge of optimal time for early infant diagnosis of HIV. Reasons for not accessing EID included fear of finding out that their child is HIV positive, feelings of guilt and/or shame and embarrassment with respect to raising an HIV infected infant. Personal experiences of HIV diagnosis and HAART were associated with participants’ attitudes and beliefs toward care-seeking behaviours. Stigma resulting from their own disclosure to others reduced their likelihood of recommending EID to other members of their communities.

**Conclusion:**

Despite the good knowledge of mothers about infant HIV infection and the availability of treatment, the knowledge of the optimal time for early infant diagnosis is lacking. Fear of infant HIV diagnosis and stigma are challenges for universal coverage of early infant diagnosis in these rural communities. Hence, community education and intensive counselling of pregnant women about early infant diagnosis are urgently needed.

## Background

Mother-to-child transmission (MTCT) of HIV is one of the major challenges of the HIV epidemic in the world and especially, Sub-Saharan Africa [1, 2]. Despite the significant progress made in reducing the rate of mother-to-child transmission (MTCT) of HIV worldwide with variations across the countries [3], children accounted for 260,000 new HIV infections in 2012. The majority of these new infections occurred in the Sub-Saharan African countries [3]. There is high morbidity and mortality associated with untreated infant HIV infections [4–6]. Without ART, 52 % of the perinatally infected infants and 26 % of post-natally infected infants will die within 12 months [7].

Many studies have demonstrated unequivocally, the immunologic, virologic and clinical benefits of early initiation of HAARTS in infants [6, 8–12], providing overwhelming evidences for the WHO’s recommendations on the early diagnosis of HIV in infants [13]. The polymerase chain reaction (PCR), which targets the DNA of the HIV for positive reaction has been rolled out worldwide for diagnosis of HIV in children from birth to 18 months [14–16]. Effective paediatric HIV treatment starts with early diagnosis, prompt initiation of HAART, and frequent monitoring to ensure retention and quality of care [17]. EID provides the HIV-exposed infants with the opportunity to receive early clinical evaluation, prophylaxis for opportunistic infections and HAART [14, 16].

**Fig. 1:**
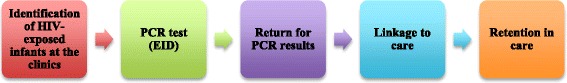
Cascade of EID Services

The major challenge facing EID services in the SSA countries is the significant rate of loss to follow up of mother-baby pair postnatally [17–22]. There is a wide variation in the completion rate (Fig. 1) from HIV testing, initiation of HAART and retention in care in African countries [17]. Report from Tanzania indicated that 88 % of infants were tested across three districts; among the infants diagnosed with HIV infection, only 69 % were enrolled in care and 39% initiated on HAART were retained in care [18]. Similar trends were observed at a tertiary hospital in South Africa, where 72 % of 838 HIV-exposed infants were tested, and only 67 % of the mothers returned for the results. Among the 38 infants diagnosed with HIV, 61 % received HAART, and only 34 % were retained in care at 68 weeks [21]. Hassan et al. (2012) in South Africa reported three-quarter of mothers dropped out of care before six months after delivery and 85 % by the twelfth month of follow up [6]. Additionally, other studies from South Africa have also, indicated poor completion rates from identification and testing of HIV-exposed infants, maternal return rate for collection of results and linkage of infected infants to care [23, 24].

EID services faces a number of programmatic challenges in Africa; migratory patterns of mothers after delivery [17]), fear of disclosure of maternal status and possible stigmatization, lack of maternal understanding of the benefits of enrolling infants in care, long waiting times at clinics and lack of transportation [25]. Stigma has been reported as a major barrier to HIV testing across Africa [26–29]. Health systems’ factors such as lack of coordination of care across HIV services resulted in delays between HIV testing, post-test counselling, and ART initiation [17].

The EID reports in South Africa, has shown that the national coverage for total PCR testing is 53.5 % (range 40.6 %-71.5 %), which is below the number expected based on antenatal HIV prevalence and live births [30]. Numerous challenges impacting on the uptake of EID were irregular /slow communication of new policy updates to relevant managers and health care workers, lack of refresher/onsite trainings, staff shortages, dried blood spot (DBS) kit supply interruption and poor DBS kit stock control [31].

The district health barometer for 2011/2012 indicated that OR Tambo district of Eastern Cape Province, South Africa had performed poorly in EID services [32]. The access of the HIV-exposed infants to the EID is dependent on the health-seeking behaviour of the mothers whose overall knowledge of the rationale and benefits of EID is the determining factor. The knowledge and attitudes of mothers toward knowing the HIV status of the child, and the willingness to accept the result warrant investigation. The valuable input from the mothers could inform future programmatic strategies for expanding access to EID in the country. The aim of the present study is to explore the perspectives and attitude of mothers towards knowing the status of their HIV-exposed infants as early as six weeks after delivery.

## Methods

### Setting

The study was conducted at two immunization clinics (Ngangeliswe and Mbekweni) chosen randomly among the primary healthcare centres in King Sabata Dalindyebo Local Municipality, OR Tambo district of Eastern Cape Province, South Africa. Ngangeliswe health centre delivers approximately one hundred and fifty babies per month with maternal HIV prevalence of 30.1 % (Unpublished local statistics, 2012). Mbekweni health centre has a large immunization clinic (average of three hundred babies per month) with similar maternal HIV prevalence. The two primary healthcare centres are located in the rural communities outside of Mthatha, providing healthcare services to people living in the deep rural communities of King Sabata Dalindyebo Local Municipality, Eastern Cape Province, South Africa. These health facilities have Nurse Initiated Management of Anti-Retroviral Therapy (NIMART) trained nurses, lay counsellors and visiting doctors, who provide care for patients with HIV at the centres. Despite the availability of EID services in both centres, the return rate for six-week PCR result was low (Unpublished local statistics, 2012).

### Design

In order to explore the maternal knowledge, concerns, fears, perceptions and attitude towards diagnosis of HIV in an infant of six weeks old, a qualitative study using semi-structure interviews, which allowed probing questions to clarify responses from participants, was considered the most appropriate design. In addition, focus group discussions were held to enrich the qualitative data, validate the findings from the interviews and to ensure group dynamics.

### Ethical approval

Ethical approval was obtained from the University of Stellenbosch Ethics Committee. The King Sabata Dalindyebo Local Municipality manager and the clinic managers of both primary healthcare centres gave permission for the study.

### Recruitment

Recruitment of the participants took place in September and October 2012. Participants were mothers whose infants had missed PCR test at six weeks (EID). Eligibility criteria were participants aged 18 years and above, had been diagnosed with HIV infection and have HIV-exposed infants less than 6 months old at the time of the study. One of the nurses at the immunization clinics purposively selected critical-case (mothers of infants who had missed the six week PCR screening) sample of eligible mothers. The objectives and methods of the study were explained to all the participants who were approached. Mothers who agreed to participate were asked to sign informed consent before the interview.

### Interview process

Semi-structure interviews with prompts were utilised to obtain qualitative data from each participant. A trained interviewer used open-ended techniques to elicit in-depth information from the participants. Each interview sessions lasted between 45 – 60 minutes. A pre-piloted interview schedule was used to ensure that the key questions were asked if they did not arise spontaneously. The interviews were conducted in the local language of the participants (isiXhosa) to ensure that participants were free and confident in their responses. The interviews were audiotaped and the notes kept.

The following knowledge and attitude toward infant HIV diagnosis were explored: mode of acquisition of HIV infection by infants, prevention of infant infections, and optimal time of screening for HIV in infants, concerns about infant with HIV infections, willingness and to test their infants. Recruitment and interim analysis were conducted after every five participants until no new information emerged during the interviews (data saturation). The results of the individual interviews were then triangulated with two focus group discussions. Eight eligible mothers from each centre participated in the focus groups. Varied individuals including those whose infants have been tested for HIV and those who were yet to test took part in the focus group discussions. The discussions were tape-recorded and field notes were taken during the sessions.

### Data analysis

The audiotaped interviews were transcribed and translated verbatim by two independent qualitative researchers. Notes were then compared to ensure accuracy of transcription and translation. Field notes were reviewed for additional information. Thematic analysis technique was used for data analysis. Line numbers were used to identify questions asked by the interviewer and responses made by the participants. Themes were developed from the participants’ responses on different questions and various issues. Participants’ responses were categorised according to themes. Themes were colour-coded and those colours were used to shade any response relating to specific themes in the interviews. Content theme analysis was employed to maximise the chance that all relevant information was grouped and coded appropriately. The notes were cross checked to ensure appropriate responses of participants.

### Data credibility

One of the authors experienced in qualitative study, independently reviewed a random sample of six interviews (including audiotapes, field notes and coding of themes) and provided feedback on the overall trustworthiness of the data and the analysis.

## Results

### Characteristics of the participants

Table 1 presents the socio-demographic characteristics of the participants according to age, level of education, marital status, employment status, total number of children (alive or dead), duration of HIV diagnosis and whether already on HAART or not. All the participants were unemployed except one participant in the individual interview. One source of their income was the child grants from the department of social services.

**Table 1:** Socio-demographic characteristics of participants

Variables	Individual interviews (*n* = 24)	Focus group discussion (*n* = 16)
Age (years)		
18–22	2 (8 %)	2 (12 %)
23–27	6 (25 %)	6 (38 %)
28–32	11 (46 %)	7 (44 %)
>32	5 (21 %)	1 (6 %)
Marital status		
Single	14 (58 %)	10 (63 %)
Married	10 (42 %)	6 (37 %)
Level of education		
< Grade 6	1 (4 %)	-
Grade 6–9	16 (67 %)	5 (31 %)
Grade 10–12	5 (21 %)	11 (69 %)
Tertiary	2 (8 %)	-
Employment status		
Employed	1 (4 %)	-
Unemployed	23 (96 %)	16 (100 %)
Total number of children (alive or dead)		
1	7 (29 %)	8 (50 %)
2	7 (29 %)	5 (31 %)
3 or more	10 (42 %)	3 (19 %)
Duration of HIV diagnosis (years)		
<1	9 (38 %)	2 (12 %)
1–3	7 (29 %)	11 (69 %)
>3	10 (33 %)	3 (19 %)
Currently on HAART		
Yes	14 (58 %)	9 (56 %)
No	10 (42 %)	7 (44 %)

*HAART* highly active antiretroviral therapy

### Themes

The following themes and sub-themes emerged from the analysis (Table 2).

**Table 2:** Summary of themes and sub-themes

Themes	Sub-themes
What do you know about HIV infection in infants?	Infection in mother can be transmitted to the child
	Time of infection during pregnancy, delivery and breastfeeding
	Window period in mother
	Other contributory factors of MTCT: low CD4 count, mixed feeding, prolonged breast feeding,
	HAART initiation in mothers
How could you prevent MTCT of HIV?	Time of initiation of HAART in pregnancy
	Adherence to treatment
	Probable risk of transmission despite HAART
Where could you obtain the infant test and when should you take your child for the test?	Time of the test
	Where the service is provided
	Time of HAART initiation in infants
If an HIV infected child is not treated, what can happen to the child?	Vulnerability to infections
	Skin changes
	Poor growth
	Poor mental development
	Death
Would you like to have HIV test on your child?	Willingness to test
	Readiness to test
	Reactions to HIV test in their children
DO you have any fears about HIV test in your child?	Reasons for fear:
What are your expectations over the waiting period for the result?	Embarrassment of having a child infected with HIV
	What people in the community might say
	Child might depend on medications to live
	Child might be falling sick
	Child might die
	Reactions towards positive results
	Confirmation of return date and willingness to collect results
Should HIV test be mandatory in the community	Benefits to other children
	Recommend HIV test to other children in your community

(1) Knowledge of infant HIV infection, (2) methods of prevention of infant HIV infection (3) HIV diagnosis (4) Effects of untreated HIV infection in infants (5) willingness and fears towards infant HIV testing, (6) expectations during waiting period and coming for results, (7) infant mandatory testing, and (8) suggestions of infant HIV test to other mothers.

#### What do you know about HIV infection in infants?

The majority of the participants demonstrated good knowledge of infant HIV infection.

*‘The infant can be infected if the mother is HIV positive and has not been tested’ (Ngangeliswe, 19, not on HAART, Individual interview).*

*“If the mother does not use pills before giving birth, the new born baby might be infected” (Mbekweni, 24, already on HAART, Individual interview).*

Some participants had misconceptions about the mode of transmission of HIV to infants.

‘*An infant is infected with HIV during birth if the mother gave birth through operation since there is a lot of blood involved’* (Mbekweni, 32, already on HAART, Focus group)

*“Touching the child by an HIV positive individual might infect the child” (Ngangeliswe, 21, not on HAART, Focus group)*

Nearly all the participants were in agreement about the risk of transmission during the time of delivery.

“*Infection happens during delivery since there is lot of blood. Also, through the blood which oozes during birth” (Mbekweni, 28, already on HAART, Individual interview)*

Few participants were aware of the risk of HIV transmission during pregnancy but had some misconceptions.

*‘The unborn child is likely to be infected since he/she shares everything with the mother’ (Ngangeliswe, 22, already on HAART, individual interview).*

Few participants were aware of the risks of HIV transmission from breast milk and the associated factors.

‘*Breastfeeding the child when you are HIV positive can lead to transmission*, but are not sure of the facts*’ (Ngangeliswe, 34, not on HAART, Focus group).*

*“Exceeding six months when breastfeeding the infant can lead to infection” (Mbekweni, 33, already on HAART, Individual interview)*

Majority of the participants considered mixed feeding as dangerous to the health of the child.

*‘The mothers must choose between breastfeeding or formula milk and not both’ (Mbekweni, 26, not on HAART, Focus group)*

*“The mother should breastfeed for six months and after that the baby can receive other foods. No water and other foods in the first six months of the child” (Ngangeliswe, 32, Already on HAART, Individual interview)*

Few individuals demonstrated fair knowledge of window period, which may give false hope that they were negative and could later infect the baby.

*‘The unborn baby is also infected as a result of the window period since the mother thinks she is negative’ (Mbekweni, 20, Not on HAART, Individual interview)*

There was an awareness of low CD4 count as an important risk factor in MTCT*.*

*‘If the CD4 count of the mother is very low, the child’s chances of infection are increased’ (Ngangeliswe, 32, Already on HAART, Individual interview)*

#### How could you prevent mother-to-child transmission of HIV?

The majority of the participants supported that women should be initiated on HAART early in pregnancy.

*‘When the mother is not treated early, the unborn baby can be infected’ (Ngangeliswe, 22, already on HAART, individual interview)*

Some participants understood that failure to adhere to ARVs is a risk for MTCT;

*“If the mother does not take treatment while being HIV positive, the infant can be infected’. If the mother starts visiting the clinic late, the infant can be infected” (Mbekweni, 28, already on HAART, Individual interview)*

The majority of the participants understood that protecting the mother from HIV infection is crucial to prevention of infant HIV infection.

*‘Parents should use condom, wear gloves when helping people in a car accident’ (Mbekweni, 24, Not on HAART, Focus group)*

Some participants demonstrated gaps in knowledge about ways to prevent infant HIV infection.

*“She knows nothing about infant infection prevention since she never got any lesson on this” (Ngangeliswe, 33, Not on HAART, Individual interview)*

There were some misconceptions demonstrated by the participants about ways to prevent infant HIV infection.

*“The child should avoid contact with other kids, there should be no sharing of children’s toothbrush” (Ngangeliswe, 19, not on HAART, Individual interview)*

#### Where can you get the HIV test and when should your child be tested?

Nearly all the participants knew that the HIV test (PCR) is offered at the clinics.

‘*We can only know the baby’s status after taking him or her to the clinic’ (Mbekweni, 24, already on HAART, Individual interview)*

Few participants knew that their babies should be tested at six weeks. However, there were variations in the times suggested by the rest of the participants.

“*The right time for the child is unknown. The child can be diagnosed at any time” (Ngangeliswe, 32, Already on HAART, Individual interview)*

Nearly all the participants did not know how soon or the right time for ARVs to be initiated in an infant with HIV infection.

*‘She doesn’t know the time of commencing ARVs, but she prefers when the child is ready’ (Mbekweni, 24, already on HAART, Individual interview)*

#### What are the symptoms of HIV infection in untreated infants?

All the participants were familiar with the symptoms of HIV infection in infants.

“*They develop rash and wounds all over the mouth and body. They usually have diarrhoea and loses energy” (Mbekweni, 24, Not on HAART, Focus group).*

Some participants reported the effect of untreated HIV infection in infants could lead to paralysis and mental disturbance. The majority of the participants knew that HIV infected infant has increased vulnerability to infections and myriads of disease conditions.

*“S/he develops TB and big tummy, as well as weight loss. Skin problems are possible with high body temperature”* (*Ngangeliswe, 33, Not on HAART, Individual interview)*

All the participants have witnessed and understood that HIV infected child can die prematurely.

### Attitude towards Early Infant HIV Diagnosis (EID)

#### Would you rather test your child for HIV now or later?

The majority of the participants never thought of bringing their babies to the clinic for HIV test.

*“She feels that her baby is too young to be tested” (Mbekweni, 24, already on HAART, Individual interview)*

*“She never thought of bringing the child to the clinic for HIV test because she was scared of injection for the little one” ((Ngangeliswe, 21, not on HAART, Focus group)*

Some of the participants who were willing to have HIV test done for their children were not prepared to do the test same day. One of the participants burst into tears when asked about her readiness for HIV test in her two-month-old boy.

*“She is not willing because the child looks HIV negative” (Ngangeliswe, 32, Already on HAART, Individual interview)*

Minority of the participants were ready to have HIV test on their infant same day.

‘*She wishes to see her child tested since she wants her child to grow like other kids and she suspects that the child might be positive” (Ngangeliswe, 22, already on HAART, individual interview)*

#### Do you have any fears about EID for your baby?

Nearly all the participants have fears about HIV test in their babies. They were worried that the result of the test might be positive. Several reasons were provided for their fears: embarrassment of having an HIV positive infants, fear of what people might say, fear that the child will depend on pills, fear of the child falling sick, fear that the child might die and fear of how she would manage to live with HIV positive child.

There were varied reactions towards the probable positive result of their infants: the majority of the participants demonstrated feeling of guilt, while some were crying and blaming themselves.

*“If the child is positive, she would be reaping what she sowed” (Mbekweni, 24, Not on HAART, Focus group)*

Few expressed numb feelings toward a positive PCR result.

#### What are your expectations over the next few days while waiting for PCR result?

Nearly all the participants accepted that there are only two possibilities from the result (either positive or negative). The majority of the participants demonstrated the tendency to believe that the results would be positive while only few expected a negative result.

*‘She expected negative and positive and wishes for the negative since she had done all that the nurses said she should do’ (Mbekweni, 24, already on HAART, Individual interview)*

Participants expressed varied reactions towards waiting for results of PCR.

“*She will feel destroyed and nervous as she will ask herself many questions. She will not even sleep at night since her baby is too young to be positive” (*Mbekweni, 32, already on HAART, Focus group)

The majority of the participants confirmed that they would return for the result on the appointment date. However, few participants doubted if they have the courage to return for the result.

*‘She will come for the result on the said date since she is curious’ (Ngangeliswe, 21, not on HAART, Focus group)*

#### Should early infant HIV be mandatory at immunization clinics?

The attitude to mandatory HIV test at six-week immunization clinics was elicited from the participants to assess the acceptability of EID. Nearly all the participant agreed that infant mandatory HIV test is beneficial to the child and parent.

*“This will help the parents so that they cannot take the child to where he shouldn’t be (traditional healer)’ (Ngangeliswe, 21, not on HAART, Focus group)*

#### Would you recommend EID to other children in your community?

The majority of the participants were cautious about recommending infant HIV test to other children in their community.

‘*Yes, since there are many mothers who do HIV test and choose not to take the results and their babies are having health problems now’ (Mbekweni, 24, already on HAART, Individual interview)*

Some participants were not keen to recommend the test to other children in their communities

‘*It is not an easy thing when you stay in the rural areas, because they think that you imply that their kids are HIV positive’ (Mbekweni, 24, Not on HAART, Focus group)*

*“I can’t do that because it requires that a person first discloses her HIV status” (Ngangeliswe, 21, not on HAART, Focus group)*

Some participants suggested that education on early infant diagnosis should be provided during antenatal period.

## Discussion

The present study found high level of awareness of mother-to-child transmission (MTCT) of HIV among the participants. The participants understood that MTCT of HIV occurs during pregnancy, delivery and breastfeeding. Previous studies reported high level of awareness about infant HIV infection in Ghana and Ethiopia, respectively [33, 34]. This finding further confirmed the effectiveness of task shifting of HIV care from doctors to nurses [35]. The NIMART initiative was introduced in South Africa to support the de-centralisation of HIV care to the primary healthcare facilities [36, 37]. NIMART trained nurses provide education on PMTCT at the setting of this study. Though, not all mothers knew the significance of the window period in relation to the risk of MTCT. This is an important phenomenon in the viral kinetics, which could pose a challenge to elimination of mother-to-child HIV transmission. The South African Guideline emphasise repeat of HIV test at 34 weeks during pregnancy [16] in order to identify those that may have sero-converted during pregnancy. HIV test at the time of delivery and at post-natal clinics could provide answers on sero-conversion during late pregnancy and during breast-feeding period.

WHO (2010) and South African National Department of Health (2013) recommended HAART initiation in all pregnant women either as prophylaxis or for maternal health [13, 16]. The risk of MTCT is effectively reduced among women initiated on HAART [38, 39]. The study participants were aware of the benefit of ART in the prevention of MTCT. Lazarus et al. (2009) reported that mothers believed that Nevirapine prophylaxis guaranteed protection against MTCT [40]. However, some participants were not aware of the risk of MTCT despite the use of ARVs. The beneficial effect of HAART in PMTCT is associated with good adherence and viral suppression [41, 42]. Besides the ART, certain pregnancy-related factors, infant factors and obstetric factors are associated with increased risk of HIV transmissions: prolonged spontaneous rupture of membrane, artificial rupture of membrane, episiotomy, prematurity and breastfeeding [43, 44]. Future cohort studies should provide answers on the rate and time of infection in infants whose mothers were exposed to HAART.

Some participants reported non-adherence to ART as an explanation for infant HIV infection despite ART in mothers. There were still knowledge gaps among the study participants on the various aspects of PMTCT and infant diagnosis, which could be improved with integration of PMTCT/EID training during antenatal period. The majority of the participants were not certain about six-week EID services and early initiation of ART in HIV-infected infants. However, most of the participants desired to know more about HIV in infants. Hassan et al. (2012) reported that the service providers did not adequately cover EID during the antenatal and PMTCT training [6]. The participants had inadequate knowledge on early infant diagnosis, which is an expected finding. The shortage of NIMART trained nurses and the large number of patients managed by the few hands available at the rural community clinics in this setting could offer plausible explanation for the observed phenomenon.

Personal experience of living with HIV and stigma from community members were some of the reasons for fear of EID among the participants, hence, impact on the access to the service at the clinics. Stigma-related to HIV/AIDS include derogatory attitudes, beliefs and behaviours directed toward people living with the disease [45, 46] affect access to HIV testing and enrolment in care. Nearly all the participants knew that the results could either be positive or negative though they tend to hope that the result would be negative. Lazarus et al. (2009) reported, “I told myself that just like in soccer, I should prepare myself to either win or lose, positive or negative” [40].

The mention of positive result generated mixed reactions from the participants; numbness, guilt, self-blame and cries. This is similar to the findings of Lazarus et al. 2009 [40], which reported that mothers go through mental preparedness to receive the results. ‘The HIV positive infants’ mothers were ‘badly affected, upset, disappointed, really hurt, feeling sad or bad………..’. In this study, majority of the participants have not thought about HIV test in their children despite missing the six-week PCR schedule. This could be attributed to unresolved acceptance of EID. Similar trend was reported in Cote d’Ivoire where mothers moderately accepted the principle of EID and fathers’ level of acceptance was low [47].

The willingness to test does not translate to readiness to accept among the study participants. This reflects the level of fear, anxiety and other emotional reactions associated with anticipated positive HIV result [48]. The reactions to waiting for the result include being unable to sleep, feeling very nervous and asking themselves many questions about the possibilities. This finding is similar to findings from Lazarus et al. [40]. Few participants were curious to know the result of their children if they were to be tested immediately, however, some lack courage to see the result of their children. Previous studies highlighted that significant proportion of mothers do not return for results of PCR [20, 21]. Therefore, a point of care PCR or other diagnostic modalities may provide proof of concept on universal coverage of EID.

Universal coverage of EID among HIV-exposed infants could be achieved if PCR test could be offered to all infants at immunization clinics and at households, however, the participants in this study had personal reservations at recommending the test to other children in their community. Despite the high prevalence of HIV in South Africa [49] and the efforts of all stakeholders to reduce stigma against people living with HIV and AIDS, people still live in fear of being stigmatised if their infants were diagnosed with HIV. More efforts are needed to eradicate HIV/AIDS related stigma especially in the rural communities.

### Strength and limitations of the study

The qualitative design of the study does not allow for generalization of findings to other populations. However, multiple primary healthcare centres and triangulation of qualitative data gave credibility to the findings from the study. The understanding of the challenges of EID in the rural health facilities and communities in South Africa might inform future policies and innovative strategies on the expansion of access to EID. Future studies should explore the health providers’ and health authorities’ perspectives on EID in South Africa.

### Public health perspectives and clinical implications

The study highlights the challenges of universal coverage of early infant diagnosis in the rural South Africa. The understanding of these challenges might provide valuable input in designing effective programmatic strategies towards universal coverage of early infant diagnosis. Health authorities should focus on incorporating EID training into PMTCT counselling sessions at the antenatal period. PMTCT counsellors and NIMART trained nurses should be trained sufficiently in EID. This will prepare mothers toward accessing the EID after delivery. Community education on infant HIV infection could reduce stigma towards mothers of HIV-infected infants.

## Conclusion

Majority of the participants have good knowledge of HIV infection in infants. However, some of them have some misconceptions about MTCT. The knowledge of early infant HIV diagnosis and early intervention with ARVs is lacking among the participants. There is a mixed attitude to early infant diagnosis: while some people were willing to carry out EID others lack the courage to test their infants. Fear, guilt, self-blame, hopelessness and embarrassment were some of the reactions expressed towards positive PCR result of their infants. Perceived fear of stigma within the community affects access to early infant diagnosis in these rural communities. Patient empowerment, improved training of counsellors on EID and strengthening of health systems are urgently needed to ensure universal coverage of EID in the country.

## Abbreviations

ART: Antiretroviral therapy
EID: Early infant diagnosis
HAART: Highly active antiretroviral therapy
MTCT: Mother-to-child transmission
NDoH: National Department of Health
NIMART: Nurse Initiated Management of Antiretroviral Treatment
PCR: Polymerase chain reaction
PMTCT: Prevention of mother-to-child transmission

## References

[1] De Cock KM, Fowler MG, Mercier E, de Vincenzi I, Saba J, Hoff E (2000). Prevention of mother-to-child HIV transmission in resource-poor countries: translating research into policy and practice. JAMA.

[2] Sperling RS, Shapiro DE, Coombs RW, Todd JA, Herman SA, McSherry GD (1996). Maternal viral load, Zidovudine treatment, and the risk of transmission of human immunodeficiency virus type 1 from mother to infant. New Engl J Med.

[3] World Health Organization. UNAIDS: Global Report: UNAIDS report on the global AIDS epidemic. Geneva: WHO. 2010. Available at: http://www.unaids.org/globalreport/Global_report.htm.

[4] Newell M-L, Coovadia K, Cortina-Borja M, Rollins N, Gaillard P, Dabis F (2004). Mortality of infected and uninfected infants born to HIV-infected mothers in Africa: a pooled analysis. The Lancet.

[5] Chilongozi D, Wang L, Brown L, Taha T, Valentine M, Emel L, et al. Morbidity and mortality among a cohort of human immunodeficiency virus type 1-infected and uninfected pregnant women and their infants from Malawi, Zambia, and Tanzania. P Infect Dis J. 2008;27(9):808. 10.1097/INF.0b013e31817109a4 PMC2739309 18679152

[6] Hassan AS, Sakwa EM, Nabwera HM, Taegtmeyer MM, Kimutai RM, Sanders EJ (2012). Dynamics and constraints of early infant diagnosis of HIV infection in rural Kenya. AIDS Behav.

[7] Becquet R, Marston M, Dabis F, Moulton LH, Gray G, Coovadia HM (2012). Children who acquire HIV infection perinatally are at higher risk of early death than those acquiring infection through breastmilk: a meta-analysis. PloS One.

[8] Chiappini E, Galli L, Tovo P-A, Gabiano C, Gattinara GC, Guarino A (2006). Virologic, immunologic and clinical benefits from early combined antiretroviral therapy in infants with perinatal HIV-1 infection. AIDS.

[9] Violari A, Cotton MF, Gibb DM, Babiker AG, Steyn J, Madhi SA (2008). Early antiretroviral therapy and mortality among HIV-infected infants. New Engl J Med.

[10] Cotton MF, Violari A, Otwombe K, Panchia R, Dobbels E, Rabie H (2013). Early time-limited antiretroviral therapy versus deferred therapy in South African infants infected with HIV: results from the children with HIV early antiretroviral (CHER) randomised trial. Lancet.

[11] Wamalwa DC, Obimbo EM, Farquhar C, Richardson BA, Mbori-Ngacha DA, Inwani I (2007). Early response to Highly Active Antiretroviral Therapy in HIV-1-infected Kenyan children. J Acquired Immune Def Synd.

[12] Bitnum A, Samson L, Chun T-W, Kakkar F, Brophy J, Murray D (2014). Early initiation of antiretroviral therapy in HIV-1-infected newborns can achieve sustained virologic suppression with low frequency of CD4 T cells carrying HIV in peripheral blood. Clin Infect Dis.

[13] World Health Organization. Antiretroviral therapy for HIV infection in infants and children: towards universal access. 2010. World Health Organization: Geneva. Available at: http://www.who.int/hiv/pub/paediatric/paed_prelim_summary/en/indexhtml [PubMed]. 2010. 23741772

[14] Sherman GG, Stevens G, Jones SA, Horsfield P, Stevens WS. Dried blood spots improve access to HIV diagnosis and care for infants in low-resource settings. J Acquired Immune Def Synd. 2005;38(5):615–7. 10.1097/01.qai.0000143604.71857.5d 15793374

[15] Creek TL, Ntumy R, Seipone K, Smith M, Mogodi M, Smit M, et al. Successful introduction of routine opt-out HIV testing in antenatal care in Botswana. J Acquired Immune Def Synd. 2007;45(1):102–7. 10.1097/QAI.0b013e318047df88 17460473

[16] National Department of Health. The South African Antiretroviral Treatment Guidelines 2013. Available at: http://www.kznhealth.gov.za/medicine/2013_art_guidelines.pdf.

[17] Motswere-Chirwa C, Voetsch A, Lu L, Letsholathebe V, Lekone P, Machakaire E, et al. Follow-up of infants diagnosed with HIV-early infant diagnosis program, Francistown, Botswana, 2005–2012. MMWR Morbidity Mortality Weekly Rep. 2014;63(7):158–60. PMC4584761 24553200

[18] Chiduo MG, Mmbando BP, Theilgaard ZP, Bygbjerg IC, Gerstoft J, Lemnge M, et al. Early infant diagnosis of HIV in three regions in Tanzania; successes and challenges. BMC Publ Health. 2013;13(1):910. 10.1186/1471-2458-13-910 PMC3852479 24088196

[19] Binagwaho A, Mugwaneza P, Irakoze AA, Nsanzimana S, Agbonyitor M, Nutt CT, et al. Scaling up early infant diagnosis of HIV in Rwanda, 2008–2010. J Public Health Policy. 2013;34(1):2–16. 10.1057/jphp.2012.62 23191941

[20] Meyers T, Moultrie H, Naidoo K, Cotton M, Eley B, Sherman G. Challenges to pediatric HIV care and treatment in South Africa. J Infect Dis. 2007;196(Supplement 3):S474–S81. 10.1086/521116 18181697

[21] Lilian RR, Kalk E, Technau K-G, Sherman GG. Birth diagnosis of HIV infection in infants to reduce infant mortality and monitor for elimination of mother-to-child transmission. Paediatr Infect Dis J. 2013;32(10):1080–5. 10.1097/INF.0b013e318290622e 23574775

[22] Patton JC, Akkers E, Coovadia AH, Meyers TM, Stevens WS, Sherman GG. Evaluation of dried whole blood spots obtained by heel or finger stick as an alternative to venous blood for diagnosis of human immunodeficiency virus type 1 infection in vertically exposed infants in the routine diagnostic laboratory. Clin Vaccine Immunol. 2007;14(2):201–3. 10.1128/CVI.00223-06 PMC1797794 17167036

[23] Grimwood A, Fatti G, Mothibi E, Eley B, Jackson D. Progress of preventing mother-to-child transmission of HIV at primary healthcare facilities and district hospitals in three South African provinces. SAMJ: South African Medical Journal. 2012;102(2):81–3. 10.7196/samj.5294 22310438

[24] Rollins N, Little K, Mzolo S, Horwood C, Newell M-L. Surveillance of mother-to-child transmission prevention programmes at immunization clinics: the case for universal screening. AIDS. 2007;21(10):1341–7. 10.1097/QAD.0b013e32814db7d4 17545711

[25] Boender TS, Sigaloff KC, Kayiwa J, Musiime V, Calis JC, Hamers RL, et al. Barriers to initiation of pediatric HIV treatment in Uganda: a mixed-method study. AIDS Res Treatment. 2012;2012. 10.1155/2012/817506 PMC3286886 22400106

[26] Babalola S. Readiness for HIV testing among young people in northern Nigeria: the roles of social norm and perceived stigma. AIDS Behav. 2007;11(5):759–69. 10.1007/s10461-006-9189-0 17191141

[27] Pettifor A, MacPhail C, Suchindran S, Delany-Moretlwe S. Factors associated with HIV testing among public sector clinic attendees in Johannesburg, South Africa. AIDS Behav. 2010;14(4):913–21. 10.1007/s10461-008-9462-5 18931903

[28] Koku EF. Desire for, and uptake of HIV tests by Ghanaian women: the relevance of community level stigma. J Comm Health. 2011;36(2):289–99. 10.1007/s10900-010-9310-1 20838860

[29] Wolff B, Nyanzi B, Katongole G, Ssesanga D, Ruberantwari A, Whitworth J. Evaluation of a home-based voluntary counselling and testing intervention in rural Uganda. Health Policy Plan. 2005;20(2):109–16. 10.1093/heapol/czi013 15746219

[30] National Health Laboratory Service. Early diagnosis of HIV-infection in infants at 6 weeks of age by province October 2009 Versus October 2010. Report prepared by the National Health Laboratory Service. Run Date: 18/11/2010 13:43:374.

[31] Woldesenbet S, Goga AE, Jackson DJ for the SA EID study group. The South African Programme to Prevent Mother-to-Child Transmission of HIV (PMTCT): Evaluation of Systems for Early Infant Diagnosis in Primary Health Care Facilities in South Africa: Report on Results of a Situational Assessment, 2010. South African Medical Research Council. Available at: http://www.nhls.ac.za/assets/files/Situational%20%20assessment%20report%20final_10th%20Oct12.pdf.

[32] Massyn N, Day C, Barron P, Haynes R, English R, Padarath A, editors. District Health Barometer 2011/12. Durban: Health Systems Trust; March 2013. Available at: http://www.health-e.org.za/wp-content/uploads/2013/04/DHB2011_12lowres.pdf.

[33] Addo VN. Pregnant women's knowledge of and attitudes to HIV testing at Komfo Anokye Teaching Hospital, Kumasi. Ghana Med J. 2006;39(2):50–4. 10.4314/gmj.v39i2.35982 PMC1790815 17299543

[34] Jebessa S, Teka T. Knowledge and attitude towards mother to child transmission of HIV and it's prevention among post natal mothers in Tikur Anbessa and Zewditu Memorial Hospitals, Addis Ababa. Ethiop J Health Dev. 2006;19(3):211–8.

[35] Callaghan M, Ford N, Schneider H. Review A systematic review of task-shifting for HIV treatment and care in Africa. Hum Resour Health. 2010;8:8–16. 10.1186/1478-4491-8-8 PMC2873343 20356363

[36] National Department of Health. The South African antiretroviral treatment guidelines 2010. Available at: http://apps.who.int/medicinedocs/documents/s19153en/s19153en.pdf.

[37] Nyazulu JCY, Muchiri E, Mazwi S, Ratshefola M. NIMART rollout to primary healthcare facilities increases access toantiretrovirals in Johannesburg: An interrupted time series analysis. SA Med J. 2013;103(4):232–6. doi:10.7196/SAMJ.6380. 10.7196/samj.6380 23547688

[38] Ngemu E, Khayeka-Wandabwa C, Kweka EJ, Choge JK, Anino E, Ooyo-Okoth E. Effectiveness of option B highly activeantiretroviral therapy (HAART) prevention of mother-to-child transmission (PMTCT) in pregnant HIV women. BMC Res Notes. 2014;7:52. 10.1186/1756-0500-7-52 PMC3898637 24447387

[39] Kouanda S, Tougri H, Cissé M, Simporé J, Pietra V, Doulougou B, et al. Impact of maternal HAART on the prevention of mother-to-child transmission of HIV: results of an 18-month follow-up study in Ouagadougou, Burkina Faso. AIDS Care. 2010;22(7):843–50. 10.1080/09540120903499204 20635248

[40] Lazarus R, Struthers H, Violari A. Starting HIV-positive babies on antiretroviral treatment: Perspectives of mothers in Soweto, South Africa. J Paediatr Health Care. 2010;24(3):176–83. 10.1016/j.pedhc.2009.07.006 20417889

[41] Delicio AM, Milanez H, Amaral E, Morais SS, Lajos GJ, e Silva JLCP, et al. Mother-to-child transmission of humanimmunodeficiency virus in aten years period. Reprod Health J. 2011;8:35. 10.1186/1742-4755-8-35 PMC3247874 22129112

[42] Townsend CL, Cortina-Borja M, Peckam CS, Ruiter A, Lyall H, Tookey PA. Low rates of mother-to-child transmission of HIV following effective pregnancy interventions in the United Kingdom and Ireland, 2000-2006. AIDS. 2008;22:973–81. 10.1097/QAD.0b013e3282f9b67a 18453857

[43] Gouveia PAC, Silva GAP, Albuquerque MFPM. Factors associated with mother-to-child transmission of the humanimmunodeficiency virus in Pernanbucho, Brazil 2000–2009. Trop Med Int Health. 2013;18(3):276–85. 10.1111/tmi.12042 23279690

[44] Ngwende S, Gombe NT, Midzi S, Tshimanga M, Shambira G, Chadambuka A. Factors associated with HIV infection amongchildren born to mothers on the prevention of mother to child transmission programme at Chitungwiza Hospital, Zimbabwe, 2008. BMC Publ Health. 2013;13:1181. 10.1186/1471-2458-13-1181 PMC3878665 24330311

[45] Simbayi LC, Kalichman S, Strebel A, Cloete A, Henda N, Mqeketo A. Internalized stigma, discrimination, and depression among men and women living with HIV/AIDS in Cape Town South Africa. Soc Sci Med. 2007;64:1823–31. 10.1016/j.socscimed.2007.01.006 PMC4271649 17337318

[46] Parker R, Aggleton P. HIV and AIDS-related stigma and discrimination: a conceptual framework and implications for action. Soc Sci Med. 2003;57:13–24. 10.1016/s0277-9536(02)00304-0 12753813

[47] Ndondoki C, Brou H, Timite-Konan M, Oga M, Amani-Bosse C, Menan H, et al. Universal HIV screening at postnatal points of care: which public health approach for early infant diagnosis in Cote d’Ivoire? PLoS One. 2013;8:e67996.doi:10.1371/journql.pone.0067996. 10.1371/journal.pone.0067996 PMC3749176 23990870

[48] Moradmand-Badie B, Moayedi-Nia S, Foroughi M, Dejman M, Nikzad R, Akhlaghkhah M, et al. HIV Positive Patients’Experiences on Receiving an HIV Positive Test: An Iranian–Qualitative Study. Am J Epidemiol Infect Dis. 2014;2(1):47–5.

[49] Simbayi L, Shisana O, Rehle T, Onoya D, Jooste S, Zungu N, et al. South African national HIV prevalence, incidence and behaviour survey, 2012. Pretoria: Human Sciences Research Council. 2014. 10.2989/16085906.2016.1153491 27002359

